# Cannabinoids—A New Perspective in Adjuvant Therapy for Pulmonary Hypertension

**DOI:** 10.3390/ijms221810048

**Published:** 2021-09-17

**Authors:** Anna Krzyżewska, Marta Baranowska-Kuczko, Krzysztof Mińczuk, Hanna Kozłowska

**Affiliations:** 1 Department of Experimental Physiology and Pathophysiology, Medical University of Białystok, 15-222 Białystok, Poland; marta.baranowska@umb.edu.pl (M.B.-K.); k.min@op.pl (K.M.); hkozl@umb.edu.pl (H.K.); 2 Department of Clinical Pharmacy, Medical University of Białystok, 15-222 Białystok, Poland

**Keywords:** pulmonary hypertension, cannabinoids, pulmonary vessels, vasorelaxation, vasoconstricion

## Abstract

Currently, no treatment can completely cure pulmonary hypertension (PH), which can lead to right ventricular failure and, consequently, death. Therefore, searching for new therapies remains important. Increased resistance in pulmonary circulation is mainly caused by the excessive contraction and proliferation of small pulmonary arteries. Cannabinoids, a group of lipophilic compounds that all interact with cannabinoid receptors, exert a pulmonary vasodilatory effect through several different mechanisms, including mechanisms that depend on vascular endothelium and/or receptor-based mechanisms, and may also have anti-proliferative and anti-inflammatory properties. The vasodilatory effect is important in regulating pulmonary resistance, which can improve patients’ quality of life. Moreover, experimental studies on the effects of cannabidiol (plant-derived, non-psychoactive cannabinoid) in animal PH models have shown that cannabidiol reduces right ventricular systolic pressure and excessive remodelling and decreases pulmonary vascular hypertrophy and pulmonary vascular resistance. Due to the potentially beneficial effects of cannabinoids on pulmonary circulation and PH, in this work, we review whether cannabinoids can be used as an adjunctive therapy for PH. However, clinical trials are still needed to recommend the use of cannabinoids in the treatment of PH.

## 1. Introduction

Pulmonary hypertension (PH) refers to a group of clinical symptoms caused by increased blood pressure (BP) in the pulmonary circulation. According to the latest classification, PH is diagnosed, when the mean pulmonary artery pressure (mPAP) at rest is over 25 mmHg, confirmed by right-sided heart catheterization. The World Health Organization (WHO) distinguishes five groups of PH: pulmonary arterial hypertension (PAH), PH due to left-sided heart disease, PH due to chronic lung disease, chronic thromboembolic PH, and PH with unexplained and/or multifactorial pathomechanisms [1,2]. PH often leads to heart failure due to the excessive overload of the right ventricle (RV), which can result in the patient’s death [3]. The development of PH is complex, and its pathogenesis can include the dysfunction of vascular endothelial cells with the excessive contraction of the pulmonary arteries, vascular, and RV remodelling (the proliferation of muscle cells and hypertrophy), inflammation, oxidative stress, and thrombosis [4,5,6].

The current treatments for PH include phosphodiesterase type 5 (PDE-5) inhibitors (e.g., sildenafil), soluble guanylate cyclase (sGC) stimulators (riociguat), endothelin receptor antagonists (ERAs) (e.g., bosentan), prostacyclin (PGI_2_) analogues (e.g., iloprost), and prostacyclin receptor (IP) agonists (selexipag) [7]. Combination therapy has emerged as the contemporary standard of care in the treatment of PH patients who are mostly symptomatic. However, this therapy does not ensure the long-term normalization of pulmonary resistance, which is an unfavorable prognostic factor. Researchers are currently seeking drugs that not only lower pulmonary resistance, but also have anti-proliferative properties [8]. There is currently no therapy that allows patients to fully recover, and PH is still characterized by high mortality [3]. Therefore, new compounds that act on signalling pathways with documented roles in the pathomechanisms of the disease are currently being sought. The first reports on the relaxing effects of cannabinoids on isolated human pulmonary vessels raised the following question: can cannabinoids be used in the treatment of PH? [9]. Hornig [10] hypothesized that cannabinoids could become an element of PH therapy but noted that we still have too little knowledge on this subject and that further experiments are needed. In this review, based on the latest reports, we explored this hypothesis in more detail.

Cannabinoids have been exploited for centuries for recreational and medicinal purposes. When smoked, cannabinoids mainly cause changes in the central nervous system. Moreover, reports suggest that cannabinoids influence the respiratory and circulatory systems. According to the United States Code (USC), marijuana is defined as all parts of the plant *Cannabis sativa* L. var. *indica* and contains about 700 compounds, more than 100 of which are cannabinoids, such as the psychoactive delta-9-tetrahydrocannabinol (THC), non-psychoactive cannabidiol (CBD), tetrahydrocannabivarin, and cannabidivarin. It is believed that marijuana has analgesic, anticonvulsant, and anti-asthmatic properties [11]. Research on the effects of plant-derived cannabinoids (phytocannabinoids) and mammalian-organism-produced endocannabinoids (arachidonic-acid derivatives) has recently received widespread interest. It is already known that the endocannabinoid system (ECS) is upregulated in some types of hypertension, including PH [12], and that the ECS components may have anti-proliferative effects [13].

The aim of this review was to determine what vascular mechanisms are involved in cannabinoid-induced pulmonary vasodilation and what effects of cannabinoids have been observed to date during in vivo studies (including experimental PH) to produce a preliminary evaluation of the usefulness of cannabinoids in the assisted treatment of PH. Another objective of this review was to examine the evidence from experimental and human studies showing what endothelium-dependent mechanisms and/or receptors are involved in cannabinoid-mediated responses in the pulmonary vasculature, including cannabinoid receptors types 1 and 2 (CB_1_-Rs and CB_2_-Rs), historically called endothelial cannabinoid receptors (eCB-Rs), transient receptor potential vanilloids 1 and 4 (TRPV1 and TRPV4), peroxisome proliferator-activated receptors-γ (PPAR-γ), and prostanoid receptors. This review only briefly describes the effects of cannabinoids on systemic vessels, as these effects have been discussed in detail in reviews by Stanley et al. [14] and Bondarenko [15].

## 2. Cannabinoids in the Cardiopulmonary System

Cannabinoids are a group of lipophilic compounds that all interact with cannabinoid receptors (CB-Rs). The current classification of cannabinoids is based on their origin: phytocannabinoids isolated, for example, from *Cannabis sativa* L. var. *indica* (e.g., THC and CBD); compounds obtained via chemical synthesis (e.g., abnormal cannabidiol (Abn-CBD); WIN 55,212-2); and components of the ECS, such as endocannabinoids (e.g., 2-arachidonoylglycerol (2-AG), N-arachidonoylethanolamine (anandamide; AEA), and virodhamine (VIR)) and endocannabinoid-like molecules (e.g., noladin ether (2-AGE), N-arachidonoyl-L-serine (ARA-S), oleamide (ODA), and L-alpha-lysophosphatidylinositol (LPI)) [16,17]. The presence of all the components of the ECS in the lungs and pulmonary vessels of animals and humans was previously confirmed by various methods (see Table 1).

The ECS components include, for example, the classic G-protein-coupled cannabinoid receptors CB_1_-R and CB_2_-R. The presence of CB_1_-Rs in the brain, liver, reproductive system, skeletal muscles, and cardiovascular system, including pulmonary vessels, has been confirmed [12,21,22,25]. CB_2_-Rs have been found in the brain, spleen, and mainly immune system cells [12,25,26,27,28]. Cannabinoids also exert their effects through other receptors such as TRPV1, TRPV4, and PPAR-γ, as well as the G-protein-coupled orphan receptors GPR18, GPR55, and eCB-Rs which are O-1918-sensitive and have not yet been cloned [29]. Endocannabinoids are mainly produced "on demand" through the synthesis of membrane phospholipid precursors [25,30]. Enzymes from the group of diacylglycerol lipases (DAGLs)—DAGL-α and DAGL-β—participate in the synthesis of AEA and 2-AG, respectively. 2-AG is degraded in the pulmonary circulation mainly by the enzyme monoacylglycerol lipase (MAGL), and AEA is mainly degraded by fatty acid amide hydrolase (FAAH) into arachidonic acid (AA) (Table 1) [30,31,32].

Cannabinoids directly exert multidirectional effects on the vascular bed, including pulmonary vessels, through interactions with appropriate receptors and indirectly through the metabolites resulting from the degradation of (endo)cannabinoids. The degradation of endocannabinoids primarily produces AA, which is converted into eicosanoids via the cyclooxygenase (COX), lipoxygenase (LOX), and cytochrome P450 (cytP450) pathways. The COX pathway that mediates the formation of PGI_2_, prostaglandins (PG), and thromboxane A2 (TXA_2_) plays the most important role in vascular responses [33]. Moreover, Sadowska et al. [19] recently demonstrated the presence of 13 endocannabinoids and endocannabinoid-related lipids in the lungs of control and monoctrotaline (MCT)-induced PH rats. These 13 endocannabinoids were AEA, 2-AG, palmitoyl ethanolamide (PEA), oleoyl ethanolamide (OEA), stearoyl ethanolamide (SEA), inolenoyl ethanolamide (LEA), palmitoleoyl ethanolamide (POEA), N-arachidonoylglycine (NAGly), docosahexaenoyl ethanolamide (DHEA), docosatetraenoyl ethanolamide (DEA), homo-γ-linolenyl ethanolamide (HEA), linoleoylglycerol (2-LG), and eicosapentaenoyl ethanolamide (EPEA), among which OEA, SEA, HEA, DEA, 2-LG, DHEA, POEA, and EPEA were examined for the first time. To date, however, little research has explored the role of the above-mentioned endocannabinoids in the physiology and pathophysiology of the pulmonary circulation.

## 3. Effects of Cannabinoids on Systemic Vessels

The ECS is unlikely to be the main element regulating the cardiovascular parameters in physiological conditions, although it plays an important role in pathological states [29,34]. The effects of cannabinoids on blood vessels have been studied since the 1990s, and new research continues to emerge. Cannabinoids in systemic circulation cause the relaxation of the blood vessels, which was extensively described by Stanley et al., in 2014. In this review, we focus on papers published after Stanley et al. (Table 2) [14]. The relaxation induced by various cannabinoids might be dependent on the endothelium [22,35,36,37,38,39,40,41,42] and/or receptors (e.g., CB_1_-Rs) [22,35,37,38,40,41,42,43] (Table 2). The potency of individual compounds depends on the vascular bed and species. As shown in Table 2, according to the negative logarithm of the concentration causing a half-maximum effect (pEC_50_) value, methanandamide (MethAEA) [42] dilated rat mesenteric arteries (rMAs) most strongly, while the weakest effects were observed for Abn-CBD in rat retinal capillaries [44] and arachidonyl cyclopropylamide (APCA) in rat aortas [43].

## 4. The Systemic Versus Pulmonary Circulation

As mentioned above, the ECS is located in the pulmonary circulation (for reviews, see Kicman and Toczek [29] and Karpińska et al. [48]) (Table 1), and its endocannabinoid components can cause the relaxation of systemic vessels, resulting in a decrease in BP [12]. Therefore, the question emerges as to whether these components could also have hypotensive effects in the pulmonary circulation. Moreover, cannabinoids can be administered by inhalation. From a pharmacological point of view, this method of delivery could accelerate the effects of their action in the pulmonary circulation. In considering this question, attention should be paid to the similarities and differences between systemic vessels and pulmonary vessels, as such factors can affect the mechanisms of action of cannabinoids. An extremely important element in the structure of pulmonary vessels is the endothelium, which, despite being a mechanical barrier also participates in maintaining proper vascular tone through the synthesis of vasoactive compounds [49]. In PH, there is a notable change in the endothelial synthesis of compounds regulating vascular tone, with a predominance of vasoconstrictors (TXA_2_, angiotensin II (ANG II), 5-hydroxytryptamine (5-HT), and endothelin 1 (ET-1)) compared to vasodilators (nitric oxide (NO) and PGI_2_) [4]. Under normal conditions, the pulmonary circulation is a low-pressure, low-resistance, and high-volume system. One of the most important features distinguishing the systemic circulation from pulmonary circulation is the presence of a mechanism that dilates blood vessels in response to hypoxia. Systemic arteries relax with decreased oxygen concentration, while pulmonary vessels constrict in response to hypoxia and increased blood oxygenation, transporting blood to more heavily oxygenated areas. Hypoxia induces hypoxic pulmonary vasoconstriction (HPV) and a hypoxic ventilatory response [50]. If hypoxia is prolonged, as can be the case in various chronic lung diseases, the spasm is accompanied by a remodelling of the vascular system leading to an increase in pulmonary vascular resistance (PVR) and the development of PH. In addition to hypoxia, the susceptibility to develop PH can also be increased by other genetic and environmental factors, even in the absence of a hypoxic stimulus [51].

## 5. Cannabinoids Affect Pulmonary Circulation

Similarly, as in the systemic circulation, cannabinoids are also shown to have a vasodilating effect in isolated pulmonary vessels (Table 3) [9,21,22,23,24,52,53,54]. Cannabinoids show a concentration-dependent vasodilating effect in human pulmonary arteries (hPAs). As shown in Table 3, according to the pEC_50_ value in hPAs, LPI [23] has the strongest vasodilatory effect (6.4), while 2-AG [21], AEA (in the presence of AM251) [9], and VIR [53] have similar levels of potency (approximately 5) and Abn-CBD [9] is the least potent. Similar results were obtained in animal pulmonary vessels (see Table 3).

At the outset, it is worth noting that the most frequently used pulmonary vasoconstrictors (i.e., U46619 (an analogue of TXA_2_) and 5-HT (Table 3)) reflect the vasoconstrictors involved in PH’s pathophysiology (see Section 4). LPI shows the strongest vasodilatory effect, but this effect could be due to the use of phenylephrine for vasoconstriction [23]. Additionally, CBD and LPI cause a time-dependent relaxation of human pulmonary vessels. Single concentrations of CBD [22] and LPI [23] produce an initial relaxation of the vessels of about 20% after 15 min, increasing to about 70% after 120 min.

**Figure 1:**
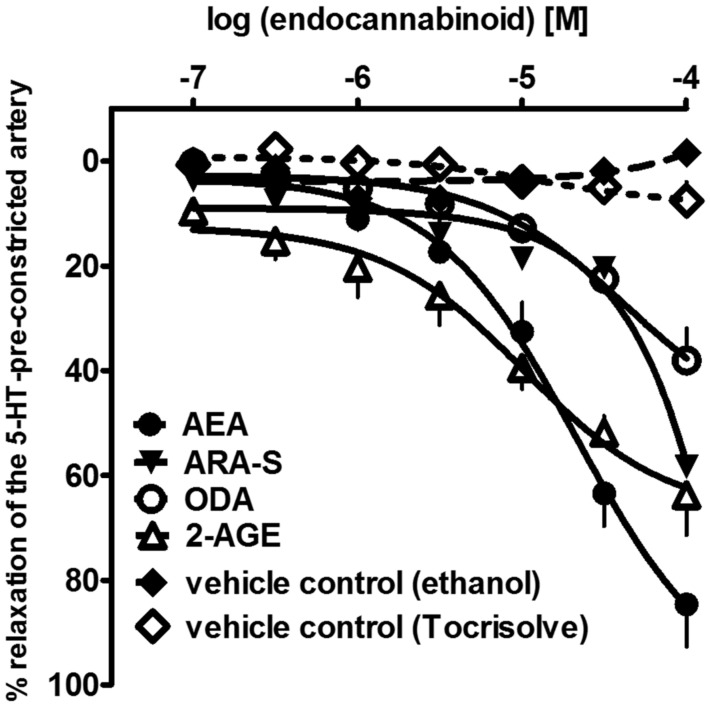
Concentration–response curves of endocannabinoid anandamide (AEA) and endocannabinoid-like molecules: N-arachidonoyl-L-serine (ARA-S), oleamide; cis-9-octadecenoamide (ODA), noladin ether; 2-arachidonyl-glycerol ether (2-AGE) or vehicles for their vasorelaxant effects on endothelium-intact rings of isolated human pulmonary artery. Results are expressed as percentage relaxation of the isometric contraction induced by serotonin (5-HT, 1 µM). Mean ± SEM of 5 tissues is shown for each curve. In few cases, SEM is smaller than or equal to the size of symbols.

In addition to the best-known endocannabinoids, in this paper, we show for the first time that the three endocannabinoid-like molecules, i.e., 2-AGE, ARA-S, and ODA, can cause a slowly developing relaxation of the endothelium-intact human pulmonary arteries (hPAs), with the following rank-order of potencies (according to their pEC_50_ values): AEA (4.8) > 2-AGE (4.6) > ARA-S (4.1) > ODA (<4) (see Figure 1). To date, the vasodilatory effects of ODA [55] and ARA-S [56] have been investigated in rMAs and aortas only. Moreover, 2-AGE was previously shown to relax rabbit pulmonary arteries [52]. 2-AGE may be an interesting focus for future studies on pulmonary arteries since oppositely to unstable 2-AG, 2-AGE does not convert to metabolites with vasoconstrictor activity in rabbit pulmonary circulation [18].

Cannabinoids cause multidirectional pulmonary vasodilatory effects mediated by the vascular endothelium and/or the COX-dependent pathway, potassium channels (i.e., calcium-activated potassium channels (K_Ca_) with small (K_Ca_2.3), intermediate (K_Ca_3.1), and large (K_Ca_1.1) conductance), cannabinoid receptors, and others (see Table 3).

## 6. Endothelium-Dependent Mechanisms of Pulmonary Vasorelaxation

In all the studies performed on isolated hPAs and animal pulmonary arteries (Table 3), the removal of the endothelium impairs vascular relaxation. This suggests the contribution of endothelium-dependent mechanisms. The removal of the vascular endothelium reduces the relaxation induced by the highest concentrations of AEA [24] and VIR [53] in hPAs by approximately 65%. The endothelium was also observed to be involved in CBD- [22], 2-AG- [21], LPI- [23], and Abn-CBD-induced relaxation [9] in hPAs. Similarly, in animal studies, the removal of the endothelium attenuates the relaxation induced by 2-AGE in rabbit pulmonary arteries [52] and by AEA or Abn-CBD in rat pulmonary arteries (rPAs) (see Table 3) [54]. In systemic vessels, endothelium denudation modifies the relaxation effect in 70% of the studies published after Stanley et al. (see Table 2) [14]. In summary, regardless of the species and vasoconstricting factors, the vascular endothelium probably plays an important role in pulmonary vasorelaxation. The mechanisms that could account for the endothelium-dependent vasodilating effects are described below (i.e., the arachidonic-acid-derived pathway, K_Ca_ channels, and the involvement of NO (see Table 3)).

### 6.1. Arachidonic-Acid-Derived Pathway

Several lines of evidence have shown that the endothelium-dependent component of cannabinoid-evoked vasorelaxation may be mediated by arachidonic-acid-derived products that occur as a result of further transformation in the COX-1/COX-2-dependent pathway [57]. The administration of URB597, an FAAH inhibitor, and indomethacin, a non-selective COX-1/COX-2 inhibitor, decreases the relaxation induced by AEA [24] and VIR [53] in hPAs. Indomethacin and nimesulide (a selective COX-2 inhibitor) inhibite the CBD-mediated relaxation of hPAs [22], suggesting the involvement of arachidonic-acid-derived metabolites in relaxation (see Table 3). Similar effects are observed in rPAs, where URB597 and indomethacin were also found to inhibit AEA-induced relaxation [54]. Some of the most important endocannabinoid-related products of the COX-1/2-dependent pathway are PGI_2_ and prostaglandins (mainly PGE_2_) [58]. PGE_2_ exerts dichotomous vascular activities and may cause vasorelaxation via the prostaglandin receptor EP_2_ or EP_4_ [22,59] and vasoconstriction via the receptor EP_1_ or EP_3_ [18]. Notably, as a result of the weakened endothelial functions in PH, the concentration of PGI_2_ decreases. By binding with its membrane receptor, PGI_2_ stimulates adenylate cyclase, which produces cyclic adenosine monophosphate (cAMP), which not only induces relaxation, but also exhibits anti-proliferative properties [60]. The involvement of IP receptors in the AEA-induced relaxation of hPAs [24] and rPAs [54] was also confirmed (Table 3). In addition to the above, it was proposed that CBD-dependent pulmonary vasodilation is mediated by the stimulation of the IP and EP_4_ receptors, as antagonists of these receptors were observed to reduce the relaxation effect (see Table 3) [22].

Notably, in an isolated mouse perfused lung model, AEA induces the contraction of the pulmonary vessels through the products of FAAH-induced AEA degradation [20]. The authors showed that AEA does not modulate vascular tone in large isolated pulmonary arteries (preconstricted with phenylephrine). In addition, hypoxia can also increase the levels of an important precursor of vasoconstrictive eicosanoids and AA in pulmonary artery smooth muscle cells (PASMCs). Moreover, the hypoxia-induced elevation of AEA and AA is restricted to PASMCs and does not occur in pulmonary endothelial cells [20]. An increase in PAP under the influence of AEA was shown in an isolated perfused rabbit lung model; the authors suggested that this increase may be related to AEA’s degradation into vasoconstricting metabolites [18], as COX-1/2-dependent-pathway metabolites might also possess vasoconstriction potency. However, more research is necessary to conclusively determine why AEA presents completely different effects between isolated vessels and the perfused lung model.

### 6.2. Vasorelaxation’s Dependence on Calcium-Dependent Potassium Channels

K_Ca_ are important in regulating pulmonary vascular tone, and impaired K_Ca_ function can lead to PH [61]. The ability of high KCl concentrations to abolish or reduce the vasorelaxation induced by cannabinoids, including AEA [24,54], VIR [53], CBD [22], and Abn-CBD [9,54], suggests the direct or indirect involvement of potassium channels (including K_Ca_). Charybdotoxin and apamin, which are K_Ca_1.1 and K_Ca_3.1 or K_Ca_2.3 inhibitors, respectively, reduce the vasorelaxant effects of Abn-CBD [9] and VIR in hPAs [53] (Table 3). This reduction may be related to the involvement of endothelium-dependent hyperpolarization (EDH) [62], which is sensitive to the combined administration of apamin and charybdotoxin in the pulmonary vasorelaxation mechanism. Iberiotoxin, which is an inhibitor of K_Ca_1.1 channels, reduces AEA-induced vasorelaxation in the human pulmonary vascular bed [24]. Similarly, iberiotoxin and TRAM-34, which are inhibitors of K_Ca_1.1 and K_Ca_3.1, respectively, significantly reduce the CBD-induced relaxation of hPA (see Table 3) [22].

In addition to the above-mentioned K_Ca_, the expression of the two-pore-domain potassium (K2P) channel was confirmed in rat and human PASMCs [63]. AEA attenuates hypoxia-induced vasoconstriction (which is one of the pathogenetic factors in PH) via the inhibition of the K2P channel in murine intra-acinar and pre-acinar arteries and does not change the vascular calibre under normoxia [63].

### 6.3. Regulation of Pulmonary Vascular Tension by NO

The incubation of isolated human pulmonary vessels with N ^G^-nitro-l-arginine methyl ester (L-NAME), an endothelial nitric oxide synthase (eNOS) inhibitor, reduces the relaxation induced by AEA [24] and, to a lesser extent, that induced by VIR [53]. A similar effect was observed for AEA in the rPA [54] (see Table 3). Notably, the NO-dependent component of AEA-evoked relaxation may be the result of direct or indirect interactions with PPAR-γ, which stimulates NO production and potentiates NO’s bioavailability [64]. In contrast to the above, it was previously shown that NO does not participate in the vasorelaxation induced by exogenous cannabinoids, especially that induced by stable analogues such as Abn-CBD [9]. Similarly, CBD-induced hPA relaxation is also NO-independent [22]. In systemic vessels, NO appears to be involved in the AEA- [38] and CBD-induced [37] relaxation of human mesenteric arteries (hMAs) (Table 2). Similarly, NAGly- [39], CBD- [46], JHW-133-, and APCA-induced [41] relaxation in rMAs was shown to be attenuated by L-NAME administration. In hMAs, L-NAME was shown to attenuate the vasodilatory effects mediated by CBD, and CBD was found to increase eNOS phosphorylation in human endothelial cells [37]. No evidence indicated the involvement of NO in Abn-CBD-mediated relaxation in rat retinal capillaries (see Table 2). These discrepancies in the mechanism of action may be due to differences between the species and structures/properties in different cannabinoid groups (endocannabinoids, phytocannabinoids, and synthetic cannabinoids such as Abn-CBD).

## 7. Receptor-Mediated Vasodilatation

It was previously suggested that the mechanisms inducing the relaxation of pulmonary vessels under the influence of cannabinoids include CB_1_-Rs/CB_2_-Rs [21], other CB receptors such as eCB-Rs [9,37,52,53,54], the cannabinoid-receptor-related orphan G-protein-coupled receptors GPR55 and GPR18 [23], and the non-cannabinoid receptors PPAR-γ [22,23], TRPV1, and TRPV4 [22]. This argument is reinforced by the fact that the presence of these receptors in the endothelium and/or smooth muscle cells was confirmed (Table 1).

### 7.1. Mechanism Dependent on CB_1_-Rs and CB_2_-Rs

The administration of the CB_1_-R antagonist AM251 and/or rimonabant attenuates 2-AG-mediated relaxation in hPAs [21], 2-AGE, and Abn-CBD-mediated relaxation in rabbit pulmonary arteries [52], suggesting the involvement of these receptors in vasodilation (see Table 3). CB_1_-R antagonists do not affect the AEA-induced relaxation of hPAs [24] or the AEA- [54], CBD- [22], and Abn-CBD-induced [54] relaxation of rPAs. Moreover, the administration of rimonabant at a concentration of 100 nM does not reduce VIR-induced relaxation, which excludes the participation of CB_1_-Rs. However, this effect was observed at a concentration of 5 μM; however, a higher concentration of rimonabant antagonizes eCB-Rs (see Table 3) [53]. Additionally, WIN 55,212-2, a synthetic agonist of CB_1_-Rs and CB_2_-Rs, does not induce the relaxation of pulmonary vessels [53].

There are indications of a previously unknown CB_1_-R-dependent endocannabinoid-mediated potential protective mechanism against excessive vasoconstriction (mainly mediated by 2-AG). AM251 attenuates 2-AG-induced vasorelaxation, indicating the involvement of CB_1_-Rs in the relaxation mechanism (see Table 3) [21]. It was suggested that vasoconstrictors such as TXA_2_ and ANG II stimulate the G_q/11_ protein, stimulating the release of 2-AG from the vascular endothelium. By acting on CB_1_-Rs, 2-AG produces vasodilation in hPAs [21,48], which may play a protective role against excessive increases in pressure in the pulmonary circulation through a so-called negative-feedback mechanism. The administration of JZL184, a MAGL inhibitor, enhances the relaxant effects of 2-AG in hPAs, suggesting that the vasorelaxant effect is caused by undegraded 2-AG, not the metabolites of 2-AG. Moreover, this experiment further confirmed that 2-AG, not AEA, is responsible for this effect, because, as described above, AEA does not act through CB_1_-Rs (see Table 3). In addition, contractions induced by U46619 in hPAs with preserved endothelium are enhanced by the presence of the DAGL inhibitor RHC80267 (responsible for the formation of 2-AG). This effect was not observed in pulmonary arteries with the endothelium removed. Experiments with RHC80267 suggested that the rapid, contractile-stimulated synthesis of 2-AG and its release from endothelial cells plays a protective role [21].

Conversely, in systemic vessels, CB_1_-Rs are involved in AEA- [38], CBD- [22,37], Meth-AEA- [42,47], and ACPA-induced relaxation [41] in human and animal mesenteric arteries (see Table 2). In systemic circulation, however, it has not yet been confirmed that the mechanism underlying the 2-AG-induced relaxation of hMAs depends on CB_1_-Rs. Moreover, it was suggested that this effect is exerted by metabolites resulting from the degradation of 2-AG in the COX-1-dependent pathway [14].

Notably, to date, no studies have confirmed the role of CB_2_-Rs in the cannabinoid-induced relaxation of isolated pulmonary vessels (Table 3) [22,24,53,54]. However, Zoratti et al. [65] demonstrated the presence of CB_2_-Rs in a calf pulmonary artery endothelial (CPAE) cell line; these CB_2_-Rs were found to be 86% homologous to the corresponding regions of the human CB_2_-R sequences. The functional analysis also showed that AEA initiates Ca^2+^ signalling in CPAE cells through the CB_2_-R activation. Although it is generally accepted that CB_2_-Rs are not directly involved in vascular relaxation [37], it was previously reported that the administration of the CB_2_-R inhibitor AM630 reduces the CBD-induced vasorelaxant effect in rat femoral arteries [45]. However, because CBD does not direclty activate CB_2_-Rs, the specific mechanism of action is unknown; it was suggested that CBD changes the function of this receptor.

### 7.2. Other G-Protein-Dependent Receptors

Previously, a cannabinoid endothelial receptor sensitive to O-1918 was considered to be a site of action for vasorelaxation. However, since this putative receptor has not yet been cloned, it remains uncertain whether it can truly be classified as a receptor. It was observed that the administration of the eCB-R antagonist O-1918 [9,53,54] reduces the AEA- [24] and VIR-induced [53], but not CBD-induced, relaxation of hPAs (see Table 3) [22]. Interestingly, three independent studies exploring the effects of Abn-CBD on the pulmonary vessels of humans [9], rabbits [52], and rats [54] suggested that this relaxation effect may depend on the presumed eCB-R, because the administration of O-1918 impairs relaxation. The administration of the pertussis toxin (PTX) (400 ng/mL, for 2 h) partially inhibits the Abn-CBD-induced vasodilation of endothelium-intact human arteries, which confirmed the involvement of a G_i_/G_o_-coupled eCB-Rs [9]. These differences in the vascular mechanisms of action of CBD and Abn-CBD, coupled with the fact that CBD is a partial agonist/antagonist of GPR18 while Abn-CBD is an agonist of GPR18, suggest that the unclassified eCB-R is probably GPR18 [15,29]. However, the putative eCB receptor antagonist may act independently of the G-protein-coupled receptors (GPCRs). Additionally, this receptor influences the functional properties of many ion channels and transporters located in the vascular system [15,66]. Reports that O-1918, after endothelial removal, also attenuates the vasodilatory effect suggest that O-1918’s site of action may be in the vascular smooth muscle [67]. Additionally, it was shown that O-1918 is an inhibitor of the Na^+^/Ca^2+^ exchanger [68] and inhibits the activity of K_Ca_1.1 channels [69], which may contribute to the regulation of vascular tone. However, this issue has not yet been unequivocally resolved in the literature [15,29,66,70].

Another receptor with confirmed expression in hPAs is GPR55, which exhibits vasorelaxant properties in the above-mentioned arteries (Table 1). LPI, an endogenous non-cannabinoid agonist of the GPR55 receptors, depending on concentration and time, causes the relaxation of isolated hPAs. The participation of GPR55 receptors in functional studies was confirmed by the use of their antagonist CID16020046, which significantly reduces the relaxation responses of hPAs stimulated with LPI [23].

### 7.3. Other G-Protein-Independent Receptors

In the pulmonary vessels, according to the current literature, only CBD causes relaxation dependent on TRPV1 receptors (see Table 3) [22]. Importantly, the presence of the TRPV1 receptors in human pulmonary vessels was confirmed (see Table 1) [22]. Capsazepine, an antagonist of TRPV1 receptors, was not observed to reduce the rPA [54] and hPA [24,53] relaxation induced by endocannabinoids such as AEA and VIR (Table 3).

The role of TRPV1 receptors in PH is unclear. Zhang et al. [71] suggested that, on the one hand, TRPV1 induces an increase in intracellular calcium ([Ca^2+^]_i_) in PASMCs and can cause vascular contractions, as well as promoting smooth muscle cell proliferation, which can lead to PH. On the other hand, the activation of TRPV1 in sensory nerves can release neuropeptides, including the calcitonin-gene-related peptide (CGRP) [71]. CGRP causes the relaxation of blood vessels and inhibits their proliferation, which may be beneficial in PH [71]. Moreover, pre-treatment with capsaicin, a specific activator of TRPV1, was found to reverse PH by alleviating inflammation [72]. Thus, the potential role of TRPV1 in PH should be further investigated.

The presence of TRPV4 receptors and their involvement in vascular relaxation mechanisms was confirmed by Addison et al. [73]. The pharmacological activation of TRPV4 receptors with the selective agonist GSK1016790A results in the relaxation of endothelium-intact rPAs preconstricted with phenylephrine [73]. In addition, the TRPV4-receptor antagonist HC067047 reduces the vasodilatory response to GSK1016790A [73,74]. Despite the above, it has not been confirmed that TRPV4 receptors participate in CBD-induced relaxation since the administration of RN1734, which antagonizes the TRPV4 receptors, does not affect relaxation [22]. Conversely, Ho et al. [36] confirmed that these receptors participate in the rMA relaxation induced by 2-AG through two antagonists, HC067047 and RN1734 (see Table 2). Moreover, TRPV4 receptors are involved in the proliferation and migration of PASMCs and may serve as a crucial target in the treatment of PH [75].

Recently, research has suggested the potential benefits of stimulating PPAR-γ receptors to alleviate PH. The PPAR-γ antagonist GW9662 reduces the time-dependent relaxation of hPAs induced by CBD (10 μM) (see Table 3) [22]. Previous studies on the potential beneficial effects of PPAR-γ receptor agonists demonstrated the PPAR-γ-receptor-mediated relaxation of human pulmonary vessels preconstricted with U46619 [76]. In addition to the above, PPAR-γ agonists exert beneficial effects on pulmonary vascular remodelling and lung morphology. Indirect evidence for the utility of PPAR-γ agonists in the treatment of PH lies in the fact that the deletion of this receptor in mouse smooth muscle [77] and endothelial cells caused the hypertrophy of the small distal pulmonary arteries and, consequently, induced PH [78]. PPAR-γ ligands interfere with the production of matrix metalloproteinases that can be activated by elastase, which was shown to prevent and reverse PH in rats. In addition to the above, PPAR-γ has anti-inflammatory properties, which include the suppression of factors related to PH, such as interleukin-6 (IL-6) and monocyte chemoattractant protein (MCP-1). PPAR-γ also protects endothelial cells against apoptosis [79]. PPAR-γ expression was found to be reduced in patients with primary and secondary PH [80], and hypoxia was found to reduce PPAR-γ expression in human pulmonary vessels [81,82]. CBD is a functional PPAR-γ agonist and was observed to cause the time-dependent relaxation of rat aortas. This effect is inhibited by the PPAR-γ antagonist GW9662, which confirms the effect of PPAR-γ on aortic relaxation [83].

## 8. Cannabinoids in PH—In Vivo and In Vitro Studies

Although the effects of cannabinoids on isolated vessels have been fairly well researched, there are still very few in vivo studies. In this review, the terms “PAH” and “PH” are reserved for human and experimental conditions, respectively [84,85]. An interesting look at the use of cannabinoids in PH therapy was presented in the latest study on CBD administration in an animal model of PH [19]. This PH model is induced in 6-to-8-week-old rats via the subcutaneous administration of 60 mg/kg MCT. The use of MCT to create an experimental model allows for the relatively simple mapping of PH in the human population by selectively damaging pulmonary vessels without adversely affecting systemic blood vessels [84]. The chronic administration of CBD as a prophylactic (see Table 4) improves blood oxygen saturation and lowers right ventricular systolic pressure (RVSP) without impacting systemic BP. CBD also reduces pulmonary arterial hypertrophy by about 30%, without any effects on RV hypertrophy [19], and normalized the plasma concentrations of plasminogen activator inhibitor-1 (PAI-1) and tissue plasminogen activator (t-PA). This effect is beneficial, because the levels of PAI-1 and t-PA are increased in PH. The above changes may partly correlate with increases in endogenous cannabinoid concentrations and AEA and NAGly in CBD-treated animals [19] (Table 4), because both endocannabinoids can relax the pulmonary [9,24,54] and systemic vessels [38,39,59] (see Table 2 and Table 3). Importantly, the chronic administration of the same dose of CBD does not change the BP or heart rate (HR) in spontaneously hypertensive rats (SHRs), rats with secondary hypertension induced by deoxycorticosterone acetate salt (DOCA salt), or their controls with normal pressure [86].

Lu et al. [87] also suggested the potential benefits of using CBD (in preventive and therapeutic models; see Table 4) in PH treatment and showed that CBD, in a preventive model, is more effective in decreasing PH phenotypes in PH mice. Mice (in a sugen-hypoxia-induced PH model) and rats (in an MCT-induced PH model) treated with CBD present lower RVSP and reduce RV and pulmonary-artery hyperproliferation. CBD also reduces the mRNA levels of inflammatory mediators such as IL-6 and tumour necrosis factor-α (TNF-α) in mouse lung tissue (see Table 4) [87]. Moreover, CBD (10 μM) was shown to inhibit the hyperproliferation of mouse PASMCs without any harmful effects on normal PASMCs. CBD was also found to recover dysfunctional mitochondria under conditions of hypoxia and relieve oxidative stress in human and mouse PASMC cell cultures. The effectiveness of CBD was also compared to that of drugs commonly used for PH, and the results suggest that CBD is as effective as bosentan or beraprost [87].

Other studies have shown an increase in PAP after the administration of AEA and 2-AG in isolated, ventilated, and buffer-perfused rabbit lungs. 2-AG showed more pronounced effects at lower concentrations. Anandamide presents a similar relationship, and an increase in PAP was observed, depending on the dose of AEA (Table 4). The authors suggested that the products from the breakdown of endocannabinoids are further metabolized to PGE_2_ and TXA_2_ (via COX-2), with vasoconstriction properties, in pulmonary arteries [18]. A similar theory was presented by Wenzel et al. [20]. According to the authors, AEA is a mediator of HPV via FAAH-dependent metabolites and is involved in the generation of PH, as discussed above.

RV failure is undoubtedly one of the worst consequences of PH. Duerr et al. [88] suggested that the ECS may play an important role in PH related to the endocannabinoid–CB_2_-R axis. In a mouse PH model induced by left pulmonary artery occlusion, researchers found that CB_2_-R-deficient (Cnr2^−/−^) mice had stronger cardiomyocytic hypertrophy and an increased Fulton’s index. The above-described effects of cannabinoids on pulmonary vascular tone and new reports on the potential beneficial effect of CBD on the animal model of PH may provide a foundation for further research. Among cannabinoids, it may be useful to explore new therapeutics for PH, especially when it is possible to create synthetic cannabinoids with selective and more concentrated actions.

## 9. Conclusions

**Figure 2:**
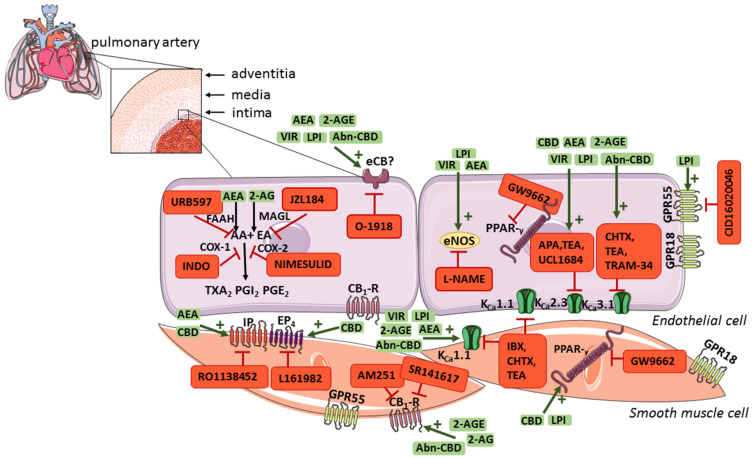
The location of the endocannabinoid system components and potential mechanisms involved in the cannabinoid-induced vasorelaxation in pulmonary arteries. Abbreviations: 2-AG, 2-arachidonoylglycerol; 2-AGE, noladin ether; AA, arachidonic acid; AEA, anandamide; Abn-CBD, abnormal cannabidiol; APA, apamin, K_Ca_2.3 inhibitor; AM251, CB_1_-R antagonist; AM630, CB_2_-R antagonist; CB_1_-R, cannabinoid receptor type 1; CB_2_-R, cannabinoid receptor type 2; CBD, cannabidiol; CHTX, charybdotoxin, K_Ca_1.1 and K_Ca_3.1 inhibitor; CID16020046, GPR55 receptor antagonist; COX-1, cyclooxygenase 1; COX-2, cyclooxygenase 2; EA, ethanolamine; eCB, historically called endothelial cannabinoid receptor; eNOS, endothelial nitric oxide synthase; EP_4_, prostanoid EP4 receptor; FAAH, fatty acid amide hydrolase; GPR18, G protein-coupled receptor 18; GPR55, G-protein-coupled receptor 55; GW9662, PPAR-γ receptor antagonist; IBX, iberiotoxin, K_Ca_1.1 inhibitor; IP, prostacyclin receptor; INDO, indometacin, COX-1/COX-2 inhibitor; JZL184, monoacylglycerol lipase inhibitor; K_Ca_2.3, K_Ca_3.1, and K_Ca_1.1, calcium-activated potassium channels with small, intermediate, and large conductivity for K^+^, respectively; LPI, l-alpha-lysophosphatidylinositol; L-NAME, N G-nitro-l-arginine methyl ester, eNOS inhibitor; L161982, EP_4_ receptor antagonist; K_ca_, calcium-activated potassium channels; MAGL, monoacylglycerol lipase; nimesulid, COX-2 inhibitor; O-1918, eCB receptor antagonist; pEC_50_, the negative logarithm of the concentration causing a half-maximum effect; PGE_2_, prostaglandin E2; PGI_2_, prostacyclin; PPAR-γ, peroxisome proliferator-activated receptor-gamma; RO1138452, IP receptor antagonist; SR141716, rimonabant, CB_1_-R antagonist; SR144528, CB_2_-R antagonist; TEA, tetraethylammonium, K_Ca_2.3 and K_Ca_3.1 inhibitor; TRAM-34, triarylmethane-34, K_Ca_2.3 inhibitor; TRPV1, transient receptor potential vanilloid 1; TXA_2_, thromboxane A2; UCL1684, 6,10-diaza-3(1,3)8,(1,4)-dibenzena-1,5(1,4)-diquinolinacy clodecaphane, K_Ca_2.3 inhibitor; URB597, FAAH inhibitor; VIR, virodhamine. This figure was prepared using a template on the Servier Medical Art website.

(Endo)cannabinoids play a role in regulating pulmonary vascular tone through endothelium-dependent and/or receptor-based mechanisms (Figure 2), which may contribute to decreasing pulmonary resistance. Moreover, the endocannabinoid negative-feedback mechanism in pulmonary arteries was found to be responsible for attenuating agonist-induced vasoconstriction, which may also play an important role in the treatment of PH. CBD, which was approved by the U.S. Food and Drug Administration and the European Medicines Agency for the treatment of drug-resistant seizures and spasticity in adult patients with multiple sclerosis, also exerts a protective effect on the vascular endothelium, decreases RVSP and/or heart remodelling and increases saturation in experimental PH, in addition to its vasorelaxant effects on pulmonary arteries. Therefore, (endo)cannabinoids represent a potential new treatment strategy as an add-on therapy for PH. Nevertheless, it should be emphasized that no clinical trials with cannabinoids in PH have yet been conducted; thus, their therapeutic potential has not been yet translated into clinical practice. In addition, single experimental studies showed that AEA and 2-AG can contract vessels and/or increase PAP. Further research, both experimental and clinical, is needed to explain these inaccuracies.

## Figures and Tables

**Table 1:** Expression of the selected components of the endocannabinoid system in pulmonary circulation/lung tissue.

Endocannabinoid System Components		Material	Species	Methods	Expression			References
					Endothelium	Whole Vascular Wall	Whole Lung	
**ligands**	2-AG	lung cellular extracts	rabbit	LC/MS			**+**	[18]
		lung	rat				**+**	[19]
	AEA	lung cellular extracts	rabbit	LC/MS			**+**	[18]
		lung	rat	LC/MS			**+**	[19]
			mouse	LC/MRM			**+**	[20]
**receptors**	CB_1_-R	pulmonary arteries	rat	IHC		**+**		[21]
				WB		**+**		
			human	WB		**+**		
				IHC		**+**		
						**+**		[22]
	CB_2_-R	pulmonary arteries	human	IHC		**+**		[22]
				WB		**+**		[21]
			rat			**+**		
	TRPV1	pulmonary arteries	human	IHC		**+**		[22]
	GPR18	pulmonary arteries	human	IHC		**+**		[22]
	GPR55	pulmonary arteries	human	WB		**+**		[23]
				IHC	**+**			
**enzymes**	FAAH	pulmonary arteries	human	WB		**+**		[24]
		lung	human	WB			**+**	
			mouse				**+**	[20]
			rabbit	RT-PCR			**+**	[18]

**+** expression detected. Abbreviations: 2-AG, 2-arachidonoylglycerol; AEA, anandamide; CB_1_-R, cannabinoid receptor type 1; CB_2_-R, cannabinoid receptor type 2; FAAH, fatty acid amide hydrolase; GPR18, G-protein-coupled receptor 18; GPR55, G-protein-coupled receptor 55; IHC, immunohistochemistry; LC/MS, liquid chromatography-mass spectrometry; LC/MRM, liquid chromatography-multiple reaction monitoring; RT-PCR, real-time polymerase chain reaction; TRPV1, transient receptor potential vanilloid 1; WB, western blot.

**Table 2:** The relaxing effects of cannabinoids on systemic vessels (published after 2014).

Ligand	Blood Vessel	pEC_50_	Mechanisms								References
			Endo	eNOS	COX	K_Ca_	CB_1_-R	CB_2_-R	eCB	Other	
**AEA**	hMA	5.7	**↓**	**↓**	**No**	**-**	**↓**	**No**	**↓**		[38]
	rRet	5.2	**-**	**-**	**-**	**-**	**-**	**-**	**-**		[40]
**2-AG**	rRet	5.0	**-**	**-**	**-**	**-**	**-**	**-**	**-**		[40]
	rMA	5.9 *	**↓**	**-**	**No**	**↓**	**No**	**No**	**-**	TRPV4	[36]
**2-AGE**	rMA	5.6 *	**No**	**-**	**-**	**-**	**-**	**-**	**-**		[36]
**NAGLy**	rMA	**-**	**↓**	**↓**	**No**	**No**	**No**	**No**	**↓**		[39]
**CBD**	hMA	5.1	**↓**	**↓**	**No**	**↓**	**↓**	**No**	**No**	TRPV1	[37]
	rFA ^1^	**-**	**No**	**↓**	**↓**	**-**	**No**	**↓**	**No**	SOD, EP_4_	[45]
	rFA, rA ^1^	**-**	**-**	**↓**	**↓**	**-**	**-**	**-**	**-**		[46]
	rMA ^1^	**-**	**-**	**No**	**No**	**-**	**-**	**-**	**-**		[46]
	rMA ^2^	6.0	**No**	**-**	**-**	**-**	**No**	**No**	**-**		[22]
	rMA ^3^	5.5	**No**	**-**	**-**	**-**	**No**	**No**	**-**		[22]
	rMA ^4^	5.9	**↓**	**-**	**-**	**-**	**↓**	**↓**	**-**		[22]
	rMA ^5^	5.6	**No**	**-**	**-**	**-**	**↓**	**No**	**-**		[22]
**Abn-CBD**	rRet	4.5	**↓**	**No**	**-**	**↓**	**No**	**No**	**-**		[44]
	pRet	**-**	**↓**	**-**	**-**	**-**	**↓**	**-**	**↓**		[35]
**WIN 55,212-2**	rRet	5.0	**↓**	**↓**	**No**	**-**	**↓**	**No**	**No**		[40]
**JHW-133**	rMA	**-**	**-**	**↓**	**-**	**-**	**-**	**↓**	**-**		[41]
**MethAEA**	rA ^6^	6.1	**-**	**-**	**-**	**-**	**-**	**-**	**-**		[47]
	rMA ^6^	4.9	**-**	**-**	**-**	**-**	**No**	**-**	**-**	TRPV1	[47]
	rMA ^4^	5.6	**-**	**-**	**-**	**-**	**↓**	**-**	**-**	TRPV1	[47]
	rMA ^5^	5.6	**-**	**-**	**-**	**-**	**↓**	**-**	**-**		[42]
	rMA ^2^	6.1	**-**	**-**	**-**	**-**	**No**	**-**	**-**		
**ACPA**	rA	4.3	**No**	**-**	**-**	**↓**	**No**	**-**	**-**	Ca_v_ 1.2	[43]
	rMA	**-**	**↓**	**↓**	**-**	**↓**	**↓**	**-**	**-**		[41]

^1^ Zucker diabetic fatty rats; ^2^ WKY, Wistar-Kyoto rats; ^3^ SHAM, control sham-operated rats; ^4^ rats with secondary hypertension induced by Deoxycorticosterone acetate-salt (DOCA salt); ^5^ SHR, spontaneously hypertensive rats; ^6^ UNX, uninephrectomized normotensive rats; * pEC_40_; **↓**, weakening effect; **No**, no effect; **-**, not determined. Abbreviations: 2-AG, 2-arachidonoylglycerol; Abn-CBD, abnormal cannabidiol; ACPA, arachidonylcyclopropylamide; AEA, anandamide; ARA-S, N-arachidonoyl L-serine; Ca_v_ 1.2, voltage-dependent L-type calcium channel subunit alpha-1C; CB_1_-R, cannabinoid receptor type 1; CB_2_-R, cannabinoid receptor type 2; CBD, cannabidiol; COX, cyclooxygenase; eCB, historically called endothelial cannabinoid receptor; endo, endothelium; eNOS, endothelial nitric oxide synthase; EP_4_, prostanoid EP_4_ receptor; FAAH, fatty acid amide hydrolase; hMA, human mesenteric artery; JHW-133, 3-(1,1-dimethylbutyl)-6aR,7,10,10aR-tetrahydro-6,6,9-trimethyl-6H-dibenzo[b,d]pyran, synthetic cannabinoid; K_Ca_, calcium-activated potassium channels; MethAEA, methanandamide; NAGLy, N-Arachidonylglycine; pEC_50_, the negative logarithm of the concentration causing a half-maximum effect; pRet, pig retinal arterioles, PTX: pertussis toxin; rA, rat aorta; rCA, rat coronary artery; rFA, rat femoral artery; rMA, rat mesenteric artery; rRet, rat retinal capillaries; SOD, superoxide dismutase; TRPV1, transient receptor potential vanilloid 1; TRPV4, transient receptor potential vanilloid 4; WIN 55,212-2, [(3R)-2,3-dihydro-5-methyl-3-(4-morpholinylmethyl)pyrrolo[1,2,3-de]-1,4-benzoxazin-6-yl]-1-naphthalenyl-methanone, monomethanesulfonate, synthetic cannabinoid; VIR, virodhamine.

**Table 3:** The vasorelaxant effects of cannabinoids on the pulmonary vessels.

Species	Ligands	Vasoconstrictor	pEC_50_	Concentration [μmol/L]	Endothelium	Inhibitors					K_Ca_ Inhibitors					Antagonists							
						eNOS	FAAH	COX-1 COX-2	COX-2	MAGL	KCl [60/120 mM]	K_Ca_1.1 K_Ca_3.1	K_Ca_1.1	K_Ca_2.3	K_Ca_3.1	CB_1_-R	CB_2_-R	eCB	IP	EP_4_	TRPV1	PPAR-γ	References
						L-NAME	URB597	INDO	NIMES	JZL184		CHTX	IBTX	UCL164/ APA *	TRAM-34	AM251/ SR141716 *	AM630/ SR144528 *	O-1918	RO1138452	L161982	CAPS	GW9662	
**human**	**AEA ^1^**	5-HT	5.2	0.1–100	**-**	**-**	-	**-**	-	-	-	-	-	-	-	**-**	-	-	-	-	-	-	[9]
	**AEA**	U46619	5.0	0.1–100	**↓**	**↓**	**↓**	**↓**	**↓**	-	**↓**	-	**↓**	-	-	**No**	**No** *	**↓**	**↓**	-	**No**	-	[24]
	**VIR**	5-HT	5.1	0.1–100	**↓**	**↓** ^3^	**↓**	**↓**	-	-	**↓**	**↓**	-	**↓** *	-	**No**	**No** *	**↓** 6.3 ^2^	-	-	**No**	-	[53]
	**2-AG**	U46619	5.4	0.01–30	**↓**	-	**No**	-	-	**↑**	**-**	-	-	-	-	**↓** 6.9 ^2^	-	-	-	-	-	-	[21]
	**LPI**	Phe	6.4	0.01–3	**↓**	**↓**	**-**	**No**	-		**↓**	-	**↓**	**↓**	**↓**	**No**	-	**↓** 5.8 ^2^	-	-	-	**↓**	[23]
	**CBD**	U46619	5.0	0.1–30	**↓**	**No**	-	**↓**	**↓**	-	**↓**	-	**↓**	**↓**	**↓**	**No**	**No**	**No**	**↓** 5.8 ^2^	**↓** 6.6 ^2^	**↓**	**↓**	[22]
	**Abn-CBD**	5-HT	4.8	0.1–100	**↓**	**No**	**-**	**No**	-	-	**↓**	**↓**	-	**↓** *	-	**-**	**-**	**↓** 5.1 ^2^	-	-	-	**-**	[9]
**rabbit**	**2-AGE**	pCa 6.3	-	0.1–3	**↓**	-	-	-	-	-	-	-	-	-	-	**↓**/ **↓** *	-	**↓**	-	-	-	-	[52]
	**Abn-CBD**	pCa 6.3	-	0.01–0.3	**↓**	-	-	-	-	-	-	-	**-**	-	-	**↓**/ **↓** *	-	**↓**	-	-	-	-	[52]
**rat**	**AEA**	U46619	5.0	0.1–100	**↓**	**↓**	**↓**	**↓**	-	-	**↓**	**↓**	-	**↓** *	-	**No**	**No**	**↓** 6.0 ^2^	**↓** 6.2 ^2^	-	**No**	-	[54]
	**Abn-CBD**	U46619	4.6	0.1–100	**↓**	**-**	-	-	-	**-**	**↓**	-	-	-	-	**No**	**No**	**↓** 5.4 ^2^	-	-	**No**	-	[54]

^1^ in the presence of AM251; ^2^ antagonistic potency (pA_2_); ^3^ statistically significant influence was noticed for virodhamine (30 μm) only; *, used antagonists; **↑**, enhancing effect; **↓**, weakening effect; **No**, no effect; **-**, not determined. Abbreviations: 2-AG, 2-arachidonoylglycerol; 2-AGE, noladin ether; 5-HT, serotonin; AEA, anandamide; Abn-CBD, abnormal cannabidiol; APA, apamin, blocker of K_Ca_2.3; AM251, CB_1_-R antagonist; AM630, CB_2_-R antagonist; CAPS, capsazepine; CB_1_-R, cannabinoid receptor type 1; CB_2_-R, cannabinoid receptor type 2; CBD, cannabidiol; CHTX, charybdotoxin, K_Ca_1.1 and K_Ca_3.1 inhibitor; COX-1, cyclooxygenase 1; COX-2, cyclooxygenase 2; eCB, historically called endothelial cannabinoid receptor; eNOS, endothelial nitric oxide synthase; EP_4_, prostanoid EP_4_ receptor; FAAH, fatty acid amide hydrolase; GW9662, 2-chloro-5-nitrobenzanilide, PPAR-γ receptor antagonist; IBX, iberiotoxin, K_Ca_1.1 inhibitor; IP, prostacyclin receptor; INDO, indometacin, COX-1/COX-2 inhibitor; JZL184, monoacylglycerol lipase inhibitor; K_Ca_, calcium-activated potassium channels; K_Ca_2.3, K_Ca_3.1, K_Ca_1.1, calcium-activated potassium channels with small, intermediate and large conductivity for K^+^, respectively; L-NAME, N G-nitro-l-arginine methyl ester, eNOS inhibitor; L-161982, EP_4_ receptor antagonist; LPI, L-alpha-lysophosphatidylinositol; MAGL, monoacylglycerol lipase; nimes: nimesulid, COX-2 inhibitor; O-1918, eCB receptor antagonist; pCa 6.3, buffer, containing free Ca^2+^ concentrations of 0.316 μM; pEC_50_, the negative logarithm of the concentration causing a half-maximum effect; Phe, phenylephrine; PPAR-γ, peroxisome proliferator-activated receptor gamma; RO1138452, IP receptor antagonist; SR141716, rimonabant, CB_1_-R antagonist; SR144528, CB_2_-R receptor antagonist; TRAM-34, triarylmethane-34, K_Ca_2.3 inhibitor; TRPV1, transient receptor potential vanilloid 1; U46619, prostanoid TP receptor agonist; UCL1684, 6,10-diaza-3(1,3)8,(1,4)-dibenzena-1,5(1,4)-diquinolinacy clodecaphane, K_Ca_2.3 inhibitor; URB597, FAAH inhibitor; VIR, virodhamine.

**Table 4:** Influence of cannabinoids on pulmonary circulation in in vivo or in vitro studies.

Species	Model	Cannabinoid	Dose/Concentration/Route of Administration	Effect	References
rabbit	isolated, ventilated, and buffer-perfused lung	AEA	0.5–5 μM	**↑** pulmonary arterial pressure	[18]
		2-AG	0.2–0.4 μM		
rat	MCT-induced PH (60 mg/kg)	CBD	10 mg/kg for 21 days, preventive model, i.p.	**↓** RVSP **↓** pulmonary arterial hypertrophy **No** right ventricular hypertrophy **↑** blood oxygen saturation **↑** concentration of endogenous cannabinoids in lung tissue: AEA, 2-LG, LEA, POEA, EPEA and NAGly **↓** the plasma concentrations of PAI-1 and t-PA	[19]
				**↓** RVSP **↓** pulmonary arterial hypertrophy **↓** right ventricular hypertrophy	[87]
mouse	sugen-hypoxia-induced PH	CBD	10 mg/kg for 21 days, preventive model, i.g.	**↓** RVSP **↓** pulmonary arterial hypertrophy **↓** right ventricular hypertrophy **↓** mRNA levels of IL-6 and TNF-α in lung tissue	[87]
			10 mg/kg for 14 days after PH induction, therapeutic model i.g.	**↓** RVSP **↓** pulmonary arterial hypertrophy **↓** right ventricular hypertrophy	
	PH-PASMC		10 μM for 2 h	**↓** hyperproliferation **↓** mRNA levels of chemokine CCL2 and CXCL10 **↓** oxidative stress in mitochondria	
human	hypoxia induced HPASMC cell culture	CBD	10 μM for 2 h and 12 h	recover the dysfunctional mitochondria in hypoxia condition: **↓** oxidative stress **↓** excessive glycolysis	[87]

**↑**, increase; **↓**, decrease; **No**, no change. Abbreviations: 2-AG, 2-arachidonoylglycerol; 2-LG, linoleoylglycerol; AEA, anandamide; CBD, cannabidiol; CCL2, monocyte chemoattractant protein-1; CXCL10, chemokine (C-X-C motif) ligand 10; EPEA, eicosapentaenoyl ethanolamide; i.g., intragastric administration; IL-6, interleukin-6; i.p., intraperitoneal injections; LEA, linolenoyl ethanolamide; NAGLy, N-arachidonoyl glycine; PH, pulmonary hypertension; PAI-1, plasminogen activator inhibitor-1; PASMCs, pulmonary artery smooth muscle cells; POEA, palmitoleoyl ethanolamide; RVSP, right ventricular systolic pressure; TNF-α, tumour necrosis factor-alpha; tPA, tissue plasminogen activator.

## Data Availability

The data presented in this study are available on request from the corresponding author. The data are not publicly available due to privacy.

## Abbreviations

2-AGE	noladin ether
Abn-CBD	abnormal-cannabidiol
APCA	arachidonylcyclopropylamide
ARA-S	arachidonoyl-L-serine
CB_1_,_2_-R	cannabinoid receptor types 1 and 2
CBD	cannabidiol
CBRs	cannabinoid receptors
DAGL-α,β	diacylglycerol lipases α,β
DHEA	docosahexaenoyl ethanolamid
DEA	docosatetraenoyl ethanolamide
ECS	endocannabinoid system
EDH	endothelium-dependent hyperpolarization
EPEA	eicosapentaenoyl ethanolamide
ERAs	endothelin receptor antagonists
HEA	homo-γ-linolenyl ethanolamide
hPAs	human pulmonary arteries
LEA	inolenoyl ethanolamide
2-LG	linoleoylglycerol
LPI L	alpha-Lysophosphatidylinositol
MCT	monocrotaline
MethAEA	methanandamide
NAGly N	arachidonoyl glycine
ODA	oleamide
OEA	oleoyl ethanolamide
PAH	pulmonary arterial hypertension
POEA	palmitoleoyl ethanolamide
PAP	pulmonary arterial pressure
PASMCs	pulmonary artery smooth muscle cells
PEA	ethanolamide
PH	pulmonary hypertension
PPAR-γ	peroxisome proliferator-activated receptor-γ
PVR	pulmonary vascular resistance
rPA	rat pulmonary artery
RVSP	right ventricular systolic pressure
SEA	stearoyl ethanolamide
U46619	analogue of thromboxane A2
VIR	virodhamine
